# Supplementary material for: CYClones: A highly powered, fully genotyped, 8-parent yeast mapping population
Source: bioRxiv. 2025 Oct 16:2025.10.15.682626. Preprint. [Version 1] doi: 10.1101/2025.10.15.682626 (PMC12632777; doi:10.1101/2025.10.15.682626)
Supplement: 1 [file NIHPP2025.10.15.682626v1-supplement-1.pdf]

bioRxiv preprint doi: https://doi.org/10.1101/2025.10.15.682626; this version posted October 16, 2025. The copyright holder for this preprint (which was not certified by peer review) is the author/funder, who has granted bioRxiv a license to display the preprint in perpetuity. It is made available under aCC-BY 4.0 International license.

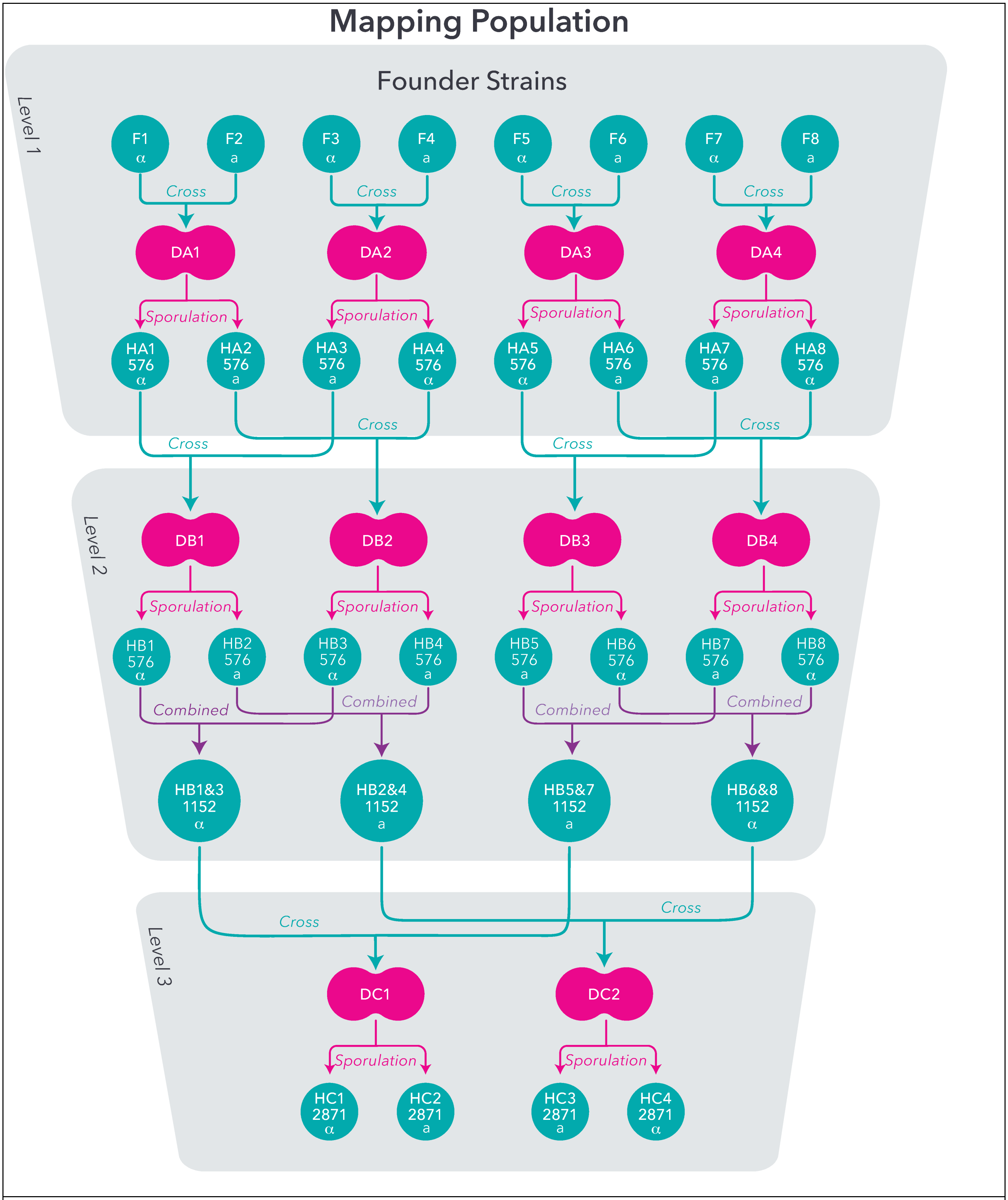

Fig S1. Detailed outline of funnel cross design. Closed shapes refer to populations or founder strains, with red indicating diploids and blue haploids. Blue lines indicate strain or population crossing, red lines indicate sporulation and purple lines indicate populations being combined. The final mapping population consists of the combined populations HC1-4.

bioRxiv preprint doi: https://doi.org/10.1101/2025.10.15.682626; this version posted October 16, 2025. The copyright holder for this preprint (which was not certified by peer review) is the author/funder, who has granted bioRxiv a license to display the preprint in perpetuity. It is made available under aCC-BY 4.0 International license.

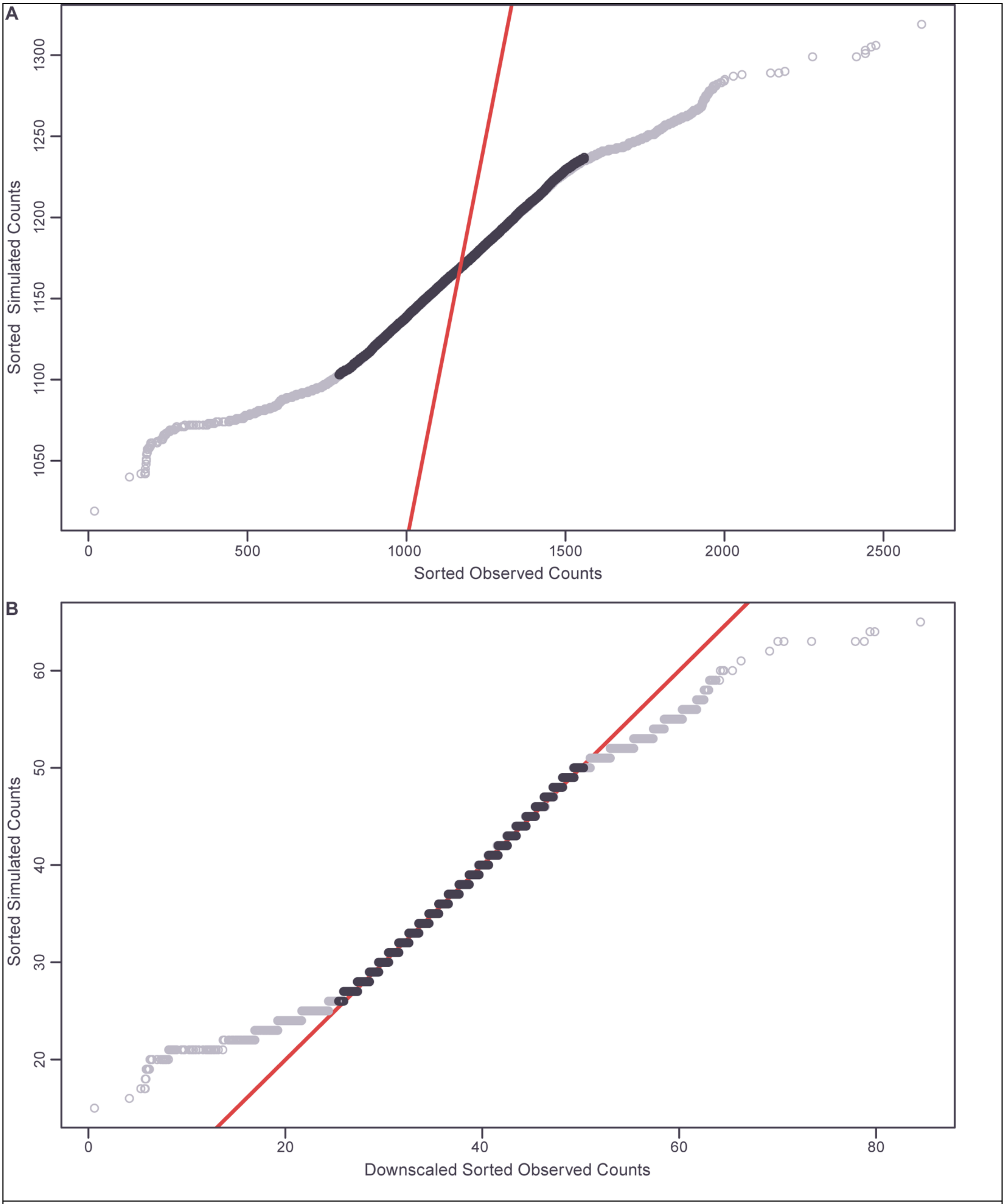

Fig S2. Q-Q plots of observed marker haplotype counts vs simulated Poisson distributions. Observed counts exclude markers on Chromosome IX. Poisson distributions generated using the same sample size and mean as the observed count distributions. Central 95% of values shown in black and x=y line in red. (**A**) Unadjusted haplotype counts. (**B**) Haplotype counts downscaled 31-fold.

bioRxiv preprint doi: https://doi.org/10.1101/2025.10.15.682626; this version posted October 16, 2025. The copyright holder for this preprint (which was not certified by peer review) is the author/funder, who has granted bioRxiv a license to display the preprint in perpetuity. It is made available under aCC-BY 4.0 International license.

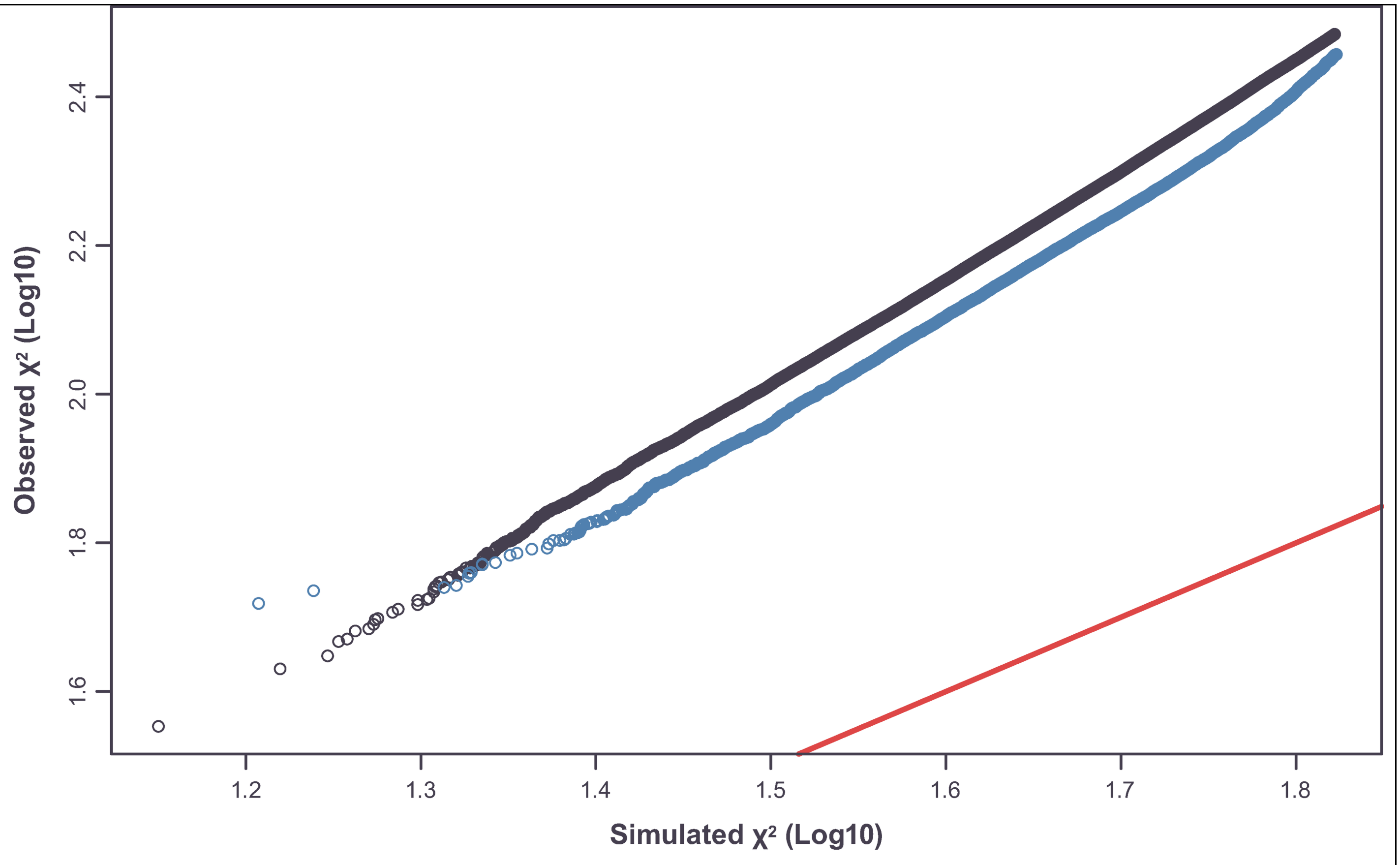

Fig S3. Q-Q plot of interchromosomal pairwise marker scores from the Chi-square test vs simulated Chi-square distribution. 20k values sampled randomly from marker pairs that include Chromosome IX (blue) and 20k values sampled randomly from marker pairs that do not include Chromosome IX (black). Simulated distribution is the same size and has the same degrees of freedom as the observed score distributions. x=y line in red. Upper 5% of scores in each distribution are excluded.

bioRxiv preprint doi: https://doi.org/10.1101/2025.10.15.682626; this version posted October 16, 2025. The copyright holder for this preprint (which was not certified by peer review) is the author/funder, who has granted bioRxiv a license to display the preprint in perpetuity. It is made available under aCC-BY 4.0 International license.

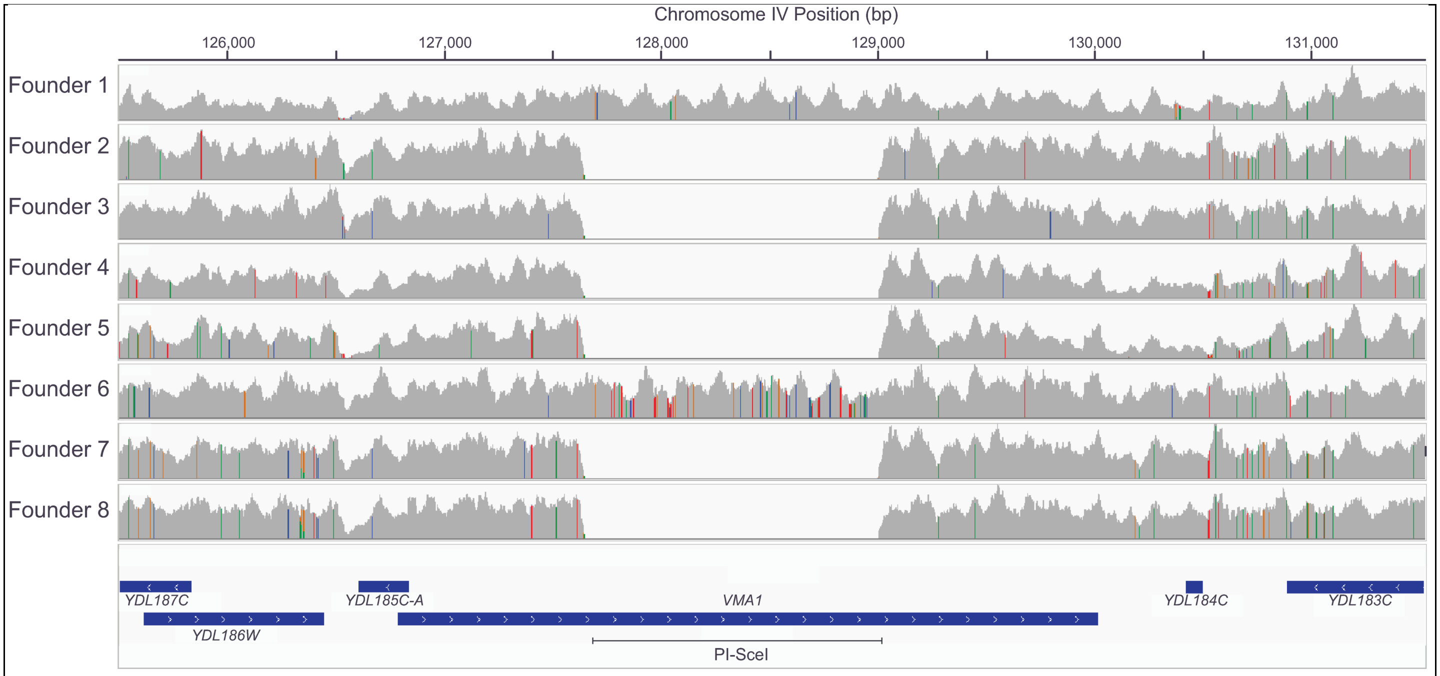

Fig S4. Presence of PI-SceI in founders 1 and 6, but not other founder strains. Visualization of read coverage (relative to the S288c reference genome) across the *VMA1* gene from Illumina sequencing of the founder strains. *VMA1* in the S288c reference genome encodes PI-SceI, with this region within *VMA1* highlighted.

bioRxiv preprint doi: https://doi.org/10.1101/2025.10.15.682626; this version posted October 16, 2025. The copyright holder for this preprint (which was not certified by peer review) is the author/funder, who has granted bioRxiv a license to display the preprint in perpetuity. It is made available under aCC-BY 4.0 International license.

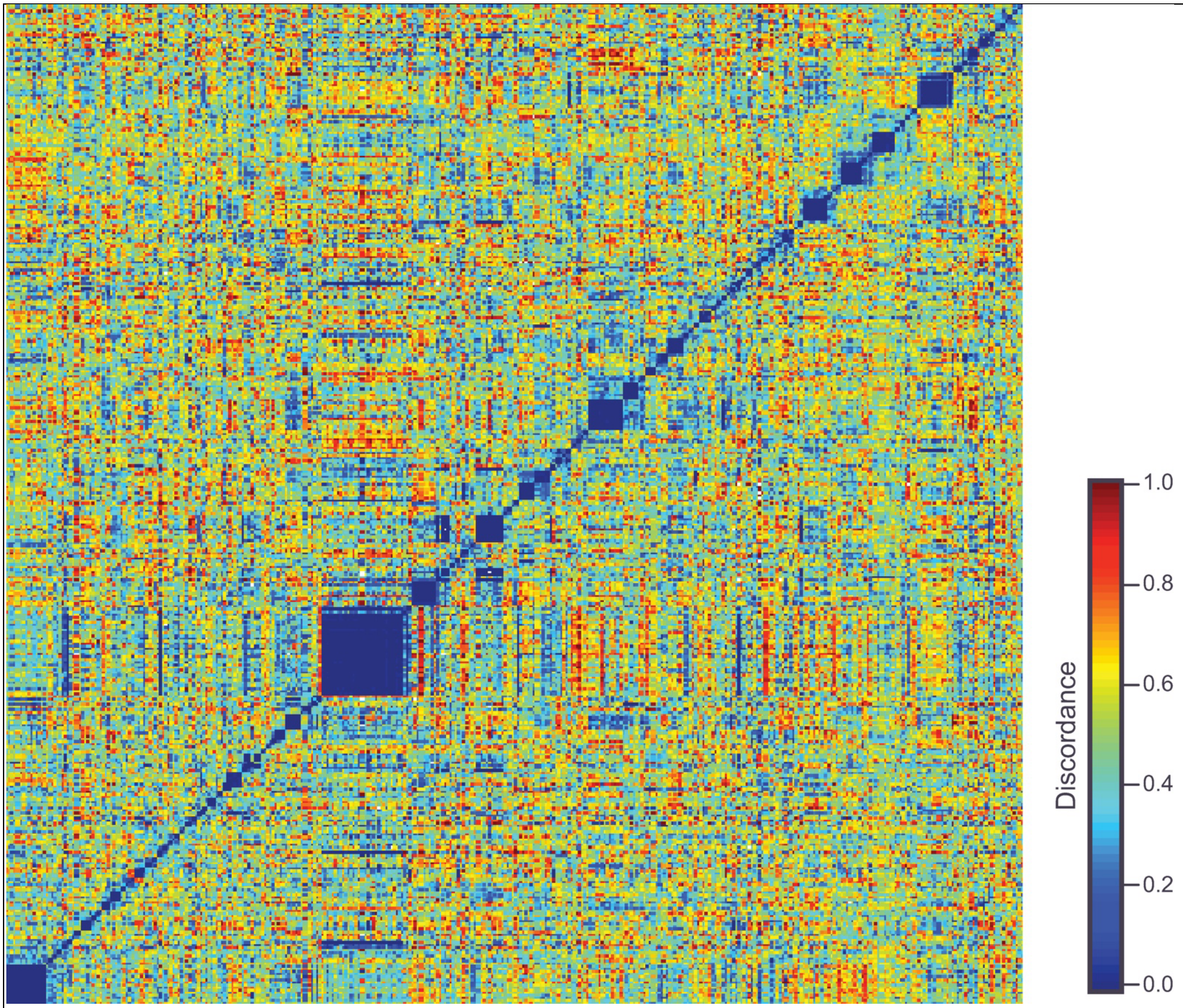

Fig S5. Quantifying shared ancestry among strains disomic for Chromosome X. Strains clustered by pairwise haplotype discordance relative to population $G_1$5-6. Distances calculated excluding markers on Chromosomes IX and X. Shared ancestry is expected to produce a discordance value of 0.
